# Adapting Translational Research Methods to Water, Sanitation, and Hygiene

**DOI:** 10.3390/ijerph16204049

**Published:** 2019-10-22

**Authors:** Karen Setty, Ryan Cronk, Shannan George, Darcy Anderson, Għanja O’Flaherty, Jamie Bartram

**Affiliations:** The Water Institute at UNC and Department of Environmental Sciences and Engineering, Gillings School of Global Public Health, University of North Carolina at Chapel Hill, 166 Rosenau Hall, CB #7431 Chapel Hill, NC 27599-7431, USA; rcronk@live.unc.edu (R.C.); swgeorge@email.unc.edu (S.G.); darcy.anderson@unc.edu (D.A.); ghanja@live.unc.edu (G.O.); jbartram@email.unc.edu (J.B.)

**Keywords:** research design, knowledge translation, evidence-based practice, implementation science, dissemination, participatory research, quality improvement

## Abstract

Translational research applies scientific techniques to achieve practical outcomes, connecting pure research and pure practice. Many translational research types have arisen since the mid-1900s, reflecting the need to better integrate scientific advancement with policy and practice. Water, sanitation, and hygiene (WaSH) development efforts have aimed to reduce morbidity and mortality and improve service delivery; thus, associated research has a strong orientation toward applied studies that use diverse methods to support decision-making. Drawing from knowledge that emerged to support other professional fields, such as manufacturing and clinical healthcare, we characterize different types of translational research and clarify nomenclature and principles. We describe study approaches relevant to translational research questions, and offer overarching recommendations, specific examples, and resources for further study as practical advice to professionals who seek to apply translational methods to WaSH problems. To enhance collective outcomes, professionals should mindfully align projects within the translational spectrum. We further recommend overarching good practices such as documenting intervention adaptations, overtly considering contextual factors, and better distinguishing efficacy from effectiveness research by replicating studies in different contexts. By consciously improving the compatibility and linkages between WaSH science and practice, this guide can accelerate urgently needed progress toward global development goals.

## 1. Introduction

Water, sanitation, and hygiene (WaSH) services underlie public health and contribute to quality of life, environmental health, social development, and economic growth. The United Nations General Assembly recognized water and sanitation as human rights in 2010 via Resolution 64/292. Since 2015, Sustainable Development Goal (SDG) 6—ensuring availability and sustainable management of water and sanitation for all by 2030—has posed a formidable and time-constrained challenge for policy, programming, and practice [1,2]. Inadequate WaSH services contribute up to 1.5% of the overall global burden of disease, with an estimated 829,000 deaths per year in low- and middle-income countries [3,4]. While earlier development policies implicitly dealt with improving access in low- and middle-income countries, the SDGs apply universally to enhancing service levels in all countries. This shift corresponds to growing recognition that WaSH disparities exist even in high-income countries and endemic disease and outbreaks persist [5,6,7,8].

Service improvements increase convenience, safety, and environmental protection. According to the World Health Organization (WHO) and United Nations Children’s Fund (UNICEF) Joint Monitoring Programme for Water Supply, Sanitation and Hygiene (JMP), water services qualify as “safely managed” when they are improved, accessible on premises, available when needed, and free from contamination [9]. Safely managed sanitation means human excreta is contained in a facility used by a single household and treated prior to safe disposal either onsite or off-site. For a variety of reasons, an estimated 29% of the global population lack water services that meet these criteria, while an estimated 61% lack safely managed sanitation [9]. Although global data on access to hygiene facilities, such as handwashing stations and soap, are not readily available, estimates suggest low coverage in many regions [9].

The combined need for “software” (i.e., behavior change and demand creation) and “hardware” (i.e., infrastructure or other technology used to deliver services) in WaSH programs often necessitates interdisciplinary collaboration. WaSH professionals come from diverse educational backgrounds, such as engineering, environmental science, public health, medicine, economics, sociology, and political science [10]. Different organizations and actors engage with research in different ways depending on their background knowledge, aims, and dominant local or broader culture. Loevinsohn et al. (2015) suggested disciplinary divisions lead actors to differently understand and evaluate WaSH programming [11]. Geographical proximity, access to learning and training resources, dissemination fora, and competitiveness among groups may further limit knowledge sharing [10].

A culture of active learning can be achieved where new knowledge (including failure) supports actionable improvement and all actors rapidly integrate it into their policies, planning, and practice. Improving access to WaSH services will require, among other things, service delivery (“supply”) from diverse professional actors as well as health-conscious behaviors (“demand”) among consumers. Effective management of the knowledge-action boundary requires professionals to actively communicate, translate, and mediate [12,13,14,15,16]. Research and researchers play a critical role in building knowledge for development, for example by identifying problems, comparing potential solutions, and devising strategies for uptake and dissemination of good practices [17]. Research addresses novel problems (e.g., emerging diseases), everyday problems (e.g., increasing efficiency), and persistent problems (e.g., delivering services to hard-to-reach populations).

Regrettably, goals, timing, and lines of communication often misalign among actor groups [18,19]. Translating novel research into everyday policy and practice can take years, and individual study recommendations may be unhelpful to decision-makers, who must consider competing needs, costs, and the prevailing political landscape [20,21]. A number of issues have chronically plagued WaSH research and development efforts, such as competition among actors for resources and visibility, bias among both researchers and publishers against publishing null results, weak political support, and inadequate human resources [2,10,22]. Recognizing pervasive challenges, some have called for greater attention to applied or “translational” research, which applies scientific techniques to address practical problems. Translational research addresses the science-to-service delivery gap [23,24], also referred to as the knowledge-to-action, research-to-practice, bench-to-bedside, or simply “death valley” distance between science and practical action [25,26]. Others note that pure research (intended to contribute to general knowledge) continues to offer value, and should not be discarded [27].

Translational research techniques, such as a guiding framework, tool, or compilation of strategies, have not yet become established or standardized within the environmental health or WaSH fields [20]. However, in recent decades, translational research structures and literature have become particularly well developed in the healthcare and mental health fields, demonstrated by active consensus and repetition in the literature. Education, pharmacy, social work, public health, and criminal justice have similarly begun to adopt translational research methods. In this case, we must rely on available examples to begin to guide professionals and harmonize meaning. Reviews aim to consolidate ideas, identify research type gaps, improve consistency, and tailor knowledge to specific fields, such as environmental sciences [20], biomedical sciences [28], or healthcare systems [29]. Hering (2018) called for a detailed mapping exercise to understand which aspects of translational science apply to the environmental domain, either directly or in a modified form [20].

Translational research traditions are supported by theories, models, tenets, and specialized language, which may evolve over time. As the boundaries of different research types are not clearly defined, terminology describing them may be reinvented, misapplied, and misused [28,29,30,31]. This may lead to confusion on which types of research are appropriate for practical problems or how to select from a plethora of methods when guidance may originate within or only apply to certain fields. Here, we define research as systematic inquiry, designed in advance, that contributes to generalizable knowledge. For simplicity, we consider all types of research falling between purely theoretical research and purely applied practice as part of a spectrum of translational research. We use “translational” as an umbrella term to span multiple research traditions aimed at converting scientific knowledge to actionable progress. We use the term “intervention” to refer broadly to any strategic change made to promote WaSH services, public health, or environmental protection within a target population and location. This could take the form of an innovative program, practice, principle, procedure, product, or policy [32].

The goals of this paper are to delineate and distill translational research principles and techniques from different fields and to discern practical advice for WaSH professionals. By examining research traditions, we aim to refine understanding of the available methods and tools and their proper application. Professionals should be able to select an appropriate translational research type and employ it to fit their purpose, context, and resource level. While many similarities and synergies exist among development needs such as health, education, and environmental protection, we limited the scope of this paper to WaSH research. The findings may aid funders looking to support research, offer clarity to decision-makers looking to interpret research, and help stakeholders better understand how to engage productively in research processes.

## 2. Methods

Since we expected few WaSH publications to align closely with existing translational science techniques or use consistent terminology, we employed a narrative review approach, which is recommended for translating research between disciplinary traditions [33]. It involves an inductive rather than exhaustive systematic or predetermined protocol, and benefits from collaborative debate. The process should remain “methodical, thorough, coherent, and manifesting a set of principles” [33]. We aimed to gather published reference material primarily from the fields of environmental science, clinical health, public health, social science, and international development using the researchers’ existing libraries and database searches. No fields were intentionally excluded.

Initially researchers contributed foundational papers describing translational research from their personal libraries. Relevant and high-quality papers that demonstrated key components of translational research were added to a group library for review and extraction of key messages. As the review proceeded, database searches using the institutional library (Articles+), Google, and Google Scholar were used to seek examples, develop a concept, or fill in gaps where literature on a given topic or representing a given research type was missing. In some cases, backward or forward citation or article similarity checks led to additional relevant publications. Specific inclusion and exclusion criteria were not developed; rather, we assessed relative readability, clarity of messaging, appropriate use of terminology, geographical and subject diversity, and novel understanding related to the phenomena under investigation.

Reviewers met biweekly over several months to discuss findings, identify trends, and develop recommendations (about 18 group sessions in total). Draft findings were first gathered and assessed qualitatively by a single team member per section using the group reference library, followed by sequential opportunities for cross-review by all other team members in both independent and interactive settings. A central part of the review involved reflexive ideation and discussion among the research team to generate consensus understanding. The team included both experienced and early career professionals, and all reviewers had research and/or work experience at other institutions in other geographies. Given the topic’s relative novelty, the team was limited to a small group familiar with translational research as well as WaSH issues. The University of North Carolina at Chapel Hill hosts both a WaSH research unit (the Water Institute) and a translational science contingent (Implementation Science Student Group, Consortium for Implementation Science, North Carolina Translational & Clinical Sciences Institute, National Implementation Research Network), furnishing opportunities for cross-pollination of concepts. External reviewers with similar expertise were identified and contacted to enhance consensus and avoid replication of effort, although few were ultimately able to participate.

Findings were grouped into six synthesis areas, as follows:

- Translational research conceptualization,
- Actor characterization,
- Translational research categorization,
- Research type comparison,
- Practical WaSH applications, and
- Overarching good practices.

Ultimately, about one-third of publications used to inform this study came from the authors’ existing libraries. By frequency, most publications came from the medical or WaSH fields, followed by publishers featuring public health or health policy; environmental science, management, or policy; science; social science; biology; international development; education; psychology; business; environmental health; epidemiology; operations; and economics topics.

## 3. Results and Discussion

### 3.1. Conceptualization of Translational Research

A broad framing of the relationship among research types can help to set the stage for researchers to identify specific approaches and methods. In 1980, the US National Institutes of Health described a research continuum, originally known as “Levy’s arrow,” that moves scientific knowledge toward a stage that reaches the public [32]. More recently, the US National Academy of Medicine developed a two-dimensional translational research continuum or “pipeline” defined by relevance and time, beginning with pre-intervention planning, followed by efficacy, effectiveness, and finally dissemination and implementation (D&I) research [32]. Within the box of D&I research, the four primary stages are “exploration, preparation (or adoption), implementation, and sustainment” [34]. A 2017 review favored a continuum conceptualization of translational research, stretched into five distinct “translational blocks” over time from pure research and early testing to effectiveness, D&I, and population-level outcomes and effectiveness [28]. These blocks (T0–T4) are commonly used as a point of reference to classify and fund medical research.

**Figure 1:**
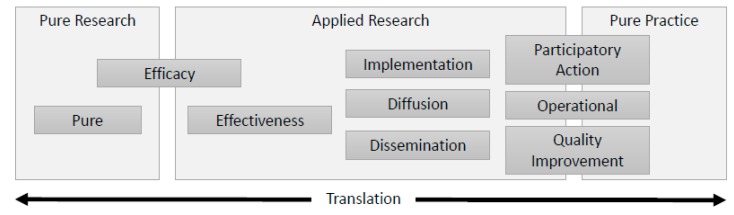
Simplified depiction of the translational research spectrum, wherein traditions have emerged distinctly but may overlap in practice.

In consolidating these concepts, we find several translational research types fall between pure research and pure practice (Figure 1). Those types to the left of Figure 1 give more attention to building scientific theory while attention to and iteration with practice increases moving toward the right of the diagram. In practice, even research at the farthest ends of each spectrum has some underlying component of practicality (e.g., to further human interests) or knowledge development (e.g., to guide future scientific investigation). Although research types are represented in Figure 1 by boxes, these borders are not strict, and positioning or alignment with any given tradition or traditions could depend on the perspective of the researcher. Some terms (e.g., “applied”) have been used as both a distinct research type and an umbrella term to describe multiple research types. We avoid using sequential time or spatial scale to define research types, because these attributes vary widely in practice. We further explore each research type according to its characteristics under “comparison of research types.”

Multi-directionality was an important supplemental concept when considering the continuum of translation. As shown in Figure 1, translation can occur in multiple directions, where practices, behaviors, and end-user communities offer information to aid research design [35]. The order of translation can begin as a supply of a research idea seeking practical application, or with a fully practical need communicated as a demand to researchers. So-called “backwards translation” increases relevance and usability by tailoring research to the values and needs of stakeholders. Further, we cannot assume linear translation, since application of scientific knowledge to practical needs may take complex and nonlinear forms [21]. Evidence and policy may be “co-produced,” as knowledge adds to and forms the basis for evolving social organization, control, identities, institutions, and discourses [36]. Research fitting different translational stages may intentionally take place in a spotty or simultaneous fashion, either at a broad scale (e.g., across different research groups) or within individual studies [37].

Another relevant concept to augment Figure 1 is the cyclical nature of translational research. Under the auspices of quality improvement, phases of the D&I process loop back using structured, repeated cycles of monitoring, evaluation, and adaptation, which helps to adapt a generic intervention to the specific setting and contextual needs [38]. These plan-do-study-act (PDSA) or plan-do-check-act (PDCA) cycles have more frequently been applied within clinical healthcare and other industries rather than WaSH services [39]. Often environmental service implementation is discussed in terms of spread or “scale-up” of interventions into new settings and contexts, where trial and evaluation take place one or more times on a limited “pilot” scale. Some traditions closer to the practice end of the spectrum (Figure 1) instead shift back and forth between research and practice stages.

### 3.2. Characterization of Actors

Lacking an established taxonomy of actors, we developed one as grounding for discussion throughout the paper (Table 1). The principal roles are intentionally broad to recognize variability and dynamism—roles may overlap or an actor may adopt additional roles. WaSH professionals come from diverse educational backgrounds and have no consistent formal certification nor qualification that would require standard training or continuing education. Many hold advanced degrees from various fields, and some may be licensed (e.g., as professional engineers). Others may be personally motivated (e.g., to advocate, financially contribute, or provide aid) and carry out research projects without formal training.

In Table 1, individual or group actors are described by their primary role(s), mission, or nature of involvement (e.g., providing services), which may apply at a range of geographical scales. The term “actor” implies active participation, while the term “stakeholder” more broadly includes anyone having a direct or indirect interest in research, whether or not they actively participate. Professionals participate in research processes in a paid (as opposed to voluntary) capacity and may also be public actors or stakeholders (e.g., if they live or volunteer in an affected service area). Decision makers include actor types such as local, regional, state, or national governments and international multilateral organizations, as defined by their means of action and capacity to issue mandatory or voluntary guidance. Actors that provide services include public, private, or cooperative utilities, and civil society organizations. Cross-disciplinary teams or networks may encompass varied roles within or across actor categories.

### 3.3. Comparison of Research Types

By comparing nomenclature, historical roots, and prevailing methods, we characterized ten loosely ordered categories of prominent research types with existing or potential relevance to WaSH (Table 2) corresponding to the translational spectrum (Figure 1). Where multiple terms are possible, we selected one but named common alternatives to promote clarity and consensus [31]. Other terms may be possible. In some instances, the terms used to describe research within a single category have very different meanings (e.g., action versus participatory) but were kept together for practicality.

#### 3.3.1. Purpose

In some cases, the purposes behind each translational research type (Table 2) relate closely enough that a research project could fall into more than one category, or researchers could blend tenets of more than one research type to achieve project goals. A common purpose for translational research is to develop evidence to inform or influence policy and practice [62]. Proponents often seek to reduce the time between when discoveries are made and changes become widespread [22]. Still, the minimum amount of time needed to translate new knowledge into practice depends on context, and acceleration could cause unintended consequences. For example, Water Safety Plans experienced a relatively rapid scale up to more than 90 countries in about 10 years [63]; however, the official launch of the term in 2004 [64] was preceded by ten years of stakeholder engagement to design an intervention that would be acceptable and feasible for diverse groups.

The degree of emphasis on practical outcomes varies by research type and project goals. Some types (e.g., effectiveness) aim to create knowledge with the underlying intent of advancing the common good, whereas others (e.g., action research and quality improvement) target specific changes as an immediate outcome (Table 2). In some cases, research might be “applied” in intent (e.g., as the motivation for conducting the work) without tangible effort toward application (e.g., if partnerships are lacking or the intervention does not yet have the face value needed to stimulate implementation).

The spatial scale of each purpose ranges from a single program, organization, or community (e.g., quality improvement and operational research) to global-scale diffusion or dissemination (Table 2). Each research type can conceptually apply at a variety of spatial scales (e.g., building, neighborhood, city, country, world) and time scales (e.g., weeks, months, years), showing they cannot be universally defined in this regard (Table 2). Increased globalization and telecommunications connectivity have made it possible to bring like-minded people who otherwise might not interact into close contact to process tasks quickly. Temporally, researchers might pursue multiple research types simultaneously to target multiple needs, or wish to prevent delays in applying interventions with strong face value by studying effectiveness of the intervention and deployment strategies simultaneously [37].

#### 3.3.2. History

While scientists have distinguished pure versus applied research since at least the 18th century, many research types arose out of necessity for innovation, for example around world wars, disease outbreaks, or widespread social movements [54]. Other research types have a theoretical basis (e.g., Rogers, 2003 [50]), where a single researcher or industry has propagated a novel viewpoint. Since science is an iterative process, some traditions (e.g., diffusion) have been renewed over decades [50]. Others have faded or evolved over time; for example, action research was a precursor of the now more widely used community-based participatory research [58,65,66].

The objectives and methods used in different translational research types sometimes differ depending on the nature (e.g., discipline or industry) of their origin (Table 2). For example, implementation science [24] and operational research [52] historically developed in relation to specific facilities (healthcare clinics and factories, respectively) and concepts may not directly carry over to other facilities or systems (e.g., water or wastewater utilities, watersheds, schools, communities), requiring tailored guidance.

Disparities in terminology may arise alongside globalization, where different nations or universities use different language for the same concept [31]. In Canada, for instance, implementation research is referred to as knowledge translation (e.g., Grimshaw et al., 2012 [62]). While some debate over terminology is beneficial and necessary as innovation progresses, the bank of terms, models, frameworks, and theories can grow excessively, leading to confusion and excessive replication of effort [67,68]. Harmonization of research efforts (when existing knowledge is accessible and sufficient) benefits translational science by making articles on similar topics available to researchers and simplifying study replication and, if needed, adaptation [31]. As capabilities for artificial intelligence grow, consistent application of terminology would aid extraction of studies from search engines and databases for systematic reviews and meta-analyses, fostering evidence synthesis [69]. For instance, the Consolidated Framework for Implementation Research (CFIR) compiled a large body of research to provide a single determinant framework and guidance on construct definitions [70].

#### 3.3.3. Actors

Regarding the actors described in Table 1 and Table 2, researchers are most often protagonists of translational research. Stakeholders frequently engage in some research types (e.g., participatory research), for instance regarding needs assessment, data elicitation, review, and communication; however, they rarely participate as drivers of other research types (e.g., efficacy trials), except as human test subjects or medical case studies. In some cases (e.g., quality improvement, action research), researchers step back to play a primarily facilitation or observational role, or service providers may step into the shoes of researchers as a way of promoting reflection and ideation. Some types of applied research occur in cooperation with service providers or are primarily carried out by service providers (e.g., action research, operations research; Baum et al., 2006 [66]).

Differences in the primary actors (Table 2) participating in translational research may stem from the underlying school of thought behind each research type. The oldest forms of pure research revolve around a single wise philosopher or scientist [71]. Newer forms of inquiry recognize that scientific research dynamics should not reinforce social and health inequities, and elevate the expertise of parties affected by the research [58]. Inclusiveness has grown through emphasis on qualitative and mixed methods that consider and integrate multiple “ways of knowing” [72,73,74]. While community stakeholder inclusion originated with participatory research traditions [57,58], including diverse stakeholders is latterly recommended for most research types. Polk (2015) and Theobald et al. (2018) suggest partnerships and co-creation of knowledge are key components of modern research [75,76].

The “participatory” nature of research can involve stakeholders to vastly different degrees (e.g., one stakeholder reviewing a final report versus a diverse stakeholder group involved in all project phases; Table 2). For instance, action and community-based participatory research traditions require explicit identification and engagement of stakeholders [58,66], while other research types simply encourage stakeholder involvement. Structured engagement, for instance in community-based participatory research, can serve as a tool for addressing equity and disparities [77]. Jiménez et al. (2019) observe that stakeholder participation is a recognized human right, and partnerships must address entrenched power structures and marginalization to avoid reinforcing and legitimizing inequities [78]. Table 1 illustrates a divide by paid (professional) and unpaid participation, which may manifest power differentials; non-professional actors are often expected to contribute with little or no compensation, because research is assumed to benefit them directly or indirectly benefit society at large [79].

Some approaches (e.g., implementation and improvement research) rely on facilitators (Table 2), requiring the corresponding human resources capacity to carry out these methods. Many researchers are technical area experts, but are not necessarily skilled at troubleshooting and customer service [38]. Implementation support may be ad hoc and fall to quickly briefed trainees rather than experienced implementation specialists. In contrast, some institutions may house specialized implementation units with more applied facilitation experience [70]. Many times in efficacy trials, costly implementation support and narrow inclusion criteria are built in to “control” fidelity to the intervention [46], making it difficult to then replicate results in translational research [80,81]. Over time, research partners may simply disappear or cease involvement given reductions in funding, shifts in activity cycles, or staff turnover, thus reducing the structural support around implementation processes over time. The specialized role of facilitation within projects and institutions may garner more attention as training in translational research methods becomes more widespread [82].

#### 3.3.4. Good Practices

Each translational research type offers tenets and methods for researchers to follow (Table 2). Methods do not necessarily define the research types, since they can be used to support multiple translational research traditions. Monitoring, program evaluation, and learning (often called “M&E” or “MEL”) could be considered tools for data gathering under most translational research types; however, some types (e.g., operations research) are more likely to emphasize quantitative methods while others (e.g., community-based participatory research) are more likely to lean on qualitative methods. Some research types require specific methods, such as selection and citation of an overarching framework for implementation research [83], application of a statistical model for operations research [84], or organization of work into timed cycles for quality [39]. Other tenets, such as sharing knowledge, generally hold true for all research types [12,85,86].

Traditional quantitative analysis tools include statistical hypothesis testing and quantitative modeling (Table 2). Over time, structured qualitative and mixed methods have become more highly valued in WaSH research. Common approaches for qualitative inquiry include ethnography, phenomenology, and grounded theory, while common tools include participant observation, interviewing, and focus groups [41]. Mixed methods research uses both qualitative and quantitative data in a planned combination or sequence to achieve the study goals [72]. Examples of qualitative and mixed WaSH research methods include user-centered design, application of economic or behavior-change theories, and social marketing.

“Confounding” (Table 2) is a complex set of identifiable and unidentifiable factors influencing delivery of evidence-based interventions. Given that context can strongly influence research outcomes [87], less-applied research types (e.g., efficacy trials) seek to control for these factors [32,46], thus avoiding bias and eliciting theoretical causality. Other research types (e.g., implementation research) embrace and describe contextual factors as a key design consideration to emphasize practical relevance [70,87].

Formal consensus on professional norms and standards of practice represents one way for good practices to become embedded in research processes over time. Clear and consistent reporting of interventions, context, and research methods is critical to advancing translational research [67,75]. Widespread adherence to tools, such as reporting checklists or templates, represents a maturation of the research tradition and offers common ground, formats, and language for users. The Equator Network maintains a repository of reporting checklists for various research types that may be useful to inform selection of appropriate guidelines (https://www.equator-network.org/), serving as a centralized resource for researchers as standards are periodically updated or new guidelines become available for additional study types.

For effectiveness and efficacy studies, reporting guidelines are well established. The CONSORT [45] and STROBE [88] guidelines provide reporting checklists for experimental and observational study designs, respectively. PRISMA guidelines specify reporting standards for systematic and scoping reviews, which may be used to synthesize evidence across multiple studies [89,90]. While SQUIRE reporting standards have been developed for quality improvement [91,92], as well as StaRI and WHO standards for implementation and operational research [93,94], they are relatively new and, as yet, little used [95]. Recent efforts to standardize specification of implementation strategies [96] will likely improve reporting over time. Development of standards for other aspects of translational research and increased application would reinforce professional consistency.

### 3.4. Model Research Questions

To help WaSH professionals reverse-design studies based on the type(s) of questions they would like to answer, we present model research questions and examples of published studies illustrating each research type (Table 3). These provide a non-exhaustive list of potential research methods useful for addressing different types of questions. Lines of inquiry for some translational research types closely relate or overlap (Table 3). For example, social networks may be explored passively as a means of observing retrospective intervention diffusion [97], or actively while prospectively intervening to disseminate the intervention [98].

### 3.5. Practical Recommendations

The following recommendations reflect key themes compiled and developed through the literature review and discussion. They represent overarching goals for incorporation into translational research activities spanning WaSH and related fields. Following a brief description, we offer a consolidated example of how each applied to a specific intervention (community-led total sanitation; Section 3.6). The recommendations and associated resources (compiled in Section 3.7) can help professionals address common barriers to effective translational research. Although not an exhaustive list of good practices, they could be used as primary considerations (e.g., as a checklist for periodic review). The recommendations may not be applicable in all situations or executable in the given order. 

#### 3.5.1. Recognize Challenges and Potential Pitfalls

As professionals, we tend to operate according to what has worked or been acceptable in the past, which has included both successes and failures. In part, undesirable but common practices continue due to a void of accountability mechanisms [119] and re-training between early career and retirement. Busy actors may fall back on meeting minimum expectations due to time or cost constraints, even when an improved practice is apparent [10].

Professional fluency in translational research methods must begin with active capacity building and access to training resources across actor types and throughout different career stages [82]. Ideally, WaSH organizations and networks would agree upon good practices and build them into day-to-day operating norms, including structures for ensuring accountability. For instance, the network Sanitation and Water for All (SWA) asks partner organizations to ascribe to a framework including guiding principles, collaborative behaviors, building blocks, and mutual accountability [120]. This approach engages multiple actor types and could help to build an enabling environment for translational research activities. Engagement and agreement could similarly be enhanced within the community of WaSH researchers, for example via the SWA research and learning constituency, International Water Association, Rural Water Supply Network, or other professional networks. A dedicated journal outlet(s) or conference event(s), existing or new, might support active dialogue on translational research for WaSH.

#### 3.5.2. Select a Guiding Theory and Define Terms

Professionals should be mindful of the placement and need for research within the translational spectrum, relative to existing knowledge. A good practice when conducting and communicating translational research is to select and reference an existing model, framework, or theory as a starting point [121]. Using a widely cited approach by default might be appropriate, although multiple options are usually available [68]. Comparing three or more theory, framework, or model options to the study characteristics or needs provides a stronger basis for justifying the selection. Birken et al. (2018) developed a user-friendly tool to help researchers select an implementation theory to fit their needs and document their reasoning [122]. Combining two or more models or frameworks within one study is possible but adds complexity and may not be desirable unless the models meet different study needs.

Researchers should appropriately identify and attribute the underlying theory when reviewing proposals or papers and ask reviewers to ensure their introductory framing is both understandable and appropriate to the study. Referencing existing work is considered a good practice to avoid rewriting lengthy methods already documented elsewhere. Pressure to demonstrate practical relevance (e.g., to funding agencies and publishers) may drive tokenistic use of terminology [27,123]. Further, some terms used interchangeably in informal English (e.g., efficacy and effectiveness) have distinct and specific meanings in translational research (Table 2). When preparing communications, authors should include references for descriptive terminology (e.g., dissemination) to clarify which research types or traditions were followed (e.g., Rabin and Brownson, 2017 [31]).

#### 3.5.3. Consider Context

The term “context” is frequently used informally within research studies, and often considered external to the project. Newer guidance specifically defines context as geographical, epidemiological, socio-cultural, socio-economic, ethical, legal, and political domains, in addition to the implementation process, actors, and the place or setting where the research occurs [70,87]. Context has received less attention outside of health sciences, such as for complex environmental interventions, where consistent understanding is in its infancy [94,124].

Context plays a critical role in research and can vastly change the interpretation of findings [125,126,127]. While researchers frequently consider prospective randomized controlled trials the gold standard for evaluating efficacy and effectiveness, consideration of contextual factors will at a minimum affect the study’s external validity. Unlike these controlled research traditions that aim to minimize confounding, translational research actively seeks to understand complicating factors and to work within the local practice context to improve applicability of findings [127]. Contextual factors frequently dictate whether an intervention is suited to a given setting or population [125,128]. Context is similarly likely to influence the intervention in less-controlled study designs such as implementation research at scale [129,130,131].

Consideration of context in translational research serves to propagate efficiency in research planning and spending. Conversely, neglect of context in research has adverse repercussions for the science-to-service gap, especially when compounded over many years [132]. For instance, an intervention proven effective in only one context might be widely embedded by policies that are later difficult to retract or worsen inequities. If an intervention is initially perceived to fail, this knowledge may be buried by publication bias [22], when the outcome is truly valuable and indicative of important contextual variables. Information about failed approaches and circumstances is highly valued in the realm of quality improvement cycles, for example, where the next trial, study, or pilot effort adjusts the implementation approach. Description of context similarly provides vital guidance for decision makers about where to deploy or scale up interventions.

A basic approach might involve preparing a structured description of context (e.g., using guidance from an applicable framework) before designing a project and again when communicating the project [91,93,94]. Qualitative or mixed methods data gathering to support structured needs assessment and scoping could involve diverse stakeholders and user-centered design techniques to aid researchers in more comprehensively identifying key contextual elements and drivers. When considered systematically throughout the project lifespan, contextual factors could influence the study design and methods, for example by guiding selection of evaluation criteria, identification of interview or focus group participants, or inclusion of influential external stakeholders.

#### 3.5.4. Involve Diverse Stakeholder Groups

Effective partnerships are critical to all translational research approaches. Cooperation and engagement are viewed as necessary to achieve the SDGs and stand at the heart of SDG targets 6.A (expand international cooperation and capacity-building support to developing countries) and 6.B (support and strengthen the participation of local communities). Partnerships often provide in-kind value by enabling increased salience, “expert” review, efficient tag-on of related research questions, and leveraging of data and resources. Actors may play different roles within research that involve varying degrees of provision or reception of knowledge [85]. Stakeholder engagement can help to design research questions, establish methods, identify synergies, and achieve practical outcomes. Active, iterative, and inclusive communication mobilizes salient, credible, and legitimate knowledge into actionable outcomes [12].

Research organizers must determine whether included stakeholders are adequate in number and sufficiently representative of the breadth of interests to optimize project goals. Organizers must also address logistical constraints (e.g., face-to-face meetings, travel funding, video conferencing software, time zone or translation accommodation) to achieve the desired degree of engagement. Partnerships rarely proceed smoothly in practice and require ongoing time investment from each party to maintain relationships and resolve problems. Logistical missteps or instincts of loyalty to one organization’s needs, culture, or work style may place partners at odds. Creating effective partnerships between researchers and service providers therefore requires conscious effort and commitment to understanding success factors and objectives, and benefits from open, frequent, and respectful communications [133]. Employers and sponsors should determine how best to directly reward communication, publishing, outreach, and networking activities.

For studies closer to pure research, partnership often consists of communication and dissemination of findings at the end stages of the project [27]. Rehfuess et al. (2016) recommend involvement of stakeholders throughout all stages of the research process to ensure study relevance and rapid implementation of findings [134]. Positive outcomes begin with inclusive and needs-responsive scoping, study design, and methods from the outset. A study in collaboration with the global SWA partnership found stakeholders were less consistently involved in the scoping and data analysis stages [10]. This could reflect the challenge of maintaining open communication across organizational silos. Alternatively, researchers might consciously exclude specific stakeholders at specific times to uphold scientific autonomy, maintain competitiveness, comply with ethical restrictions, or conserve resources.

Continued attention to conscious and strategic communication throughout projects would enhance cross-sector cooperation and boundary communication [15]. Explicitly identifying and recognizing value for each stakeholder involved and balancing their respective needs can bolster partnership success. Recognizing inequities, ethical research should entail capacity development to ultimately reduce power imbalances [17,135].

As one partnership example, a research institute (the Water Institute at UNC) and an international civil society organization (World Vision) collaborated to explore the processes that contribute to sustainability of rural community managed water supplies in multiple countries. The two groups worked together to identify a problem (i.e., characteristics of the most effective water committees) stemming from practice (i.e., of practical interest to programming providers) where little evidence was available (i.e., an interesting, novel research question for researchers). Community members (individuals, water committees, and field staff) engaged as participants through interviews, focus groups, and mapping exercises, providing a key source of data [136,137,138,139]. Through consensus-based research questions, the partners realized value toward both evidence generation and practical intervention.

#### 3.5.5. Document Fidelity and Adaptations

While many programs measure intervention outcomes, one can also measure implementation outcomes (e.g., acceptability, adoption, appropriateness, feasibility, fidelity, implementation cost, penetration or reach, and sustainability; Proctor et al., 2011 [140]), also known as process evaluation. This addresses the question, “was the intervention implemented?” in addition to the question, “did the intervention work?” Comprehensive evaluation plans that include both intervention outcomes and implementation outcomes are useful for assessing implementation fidelity [141,142]. Such measurement can avoid simplistic interpretation of an intervention as ineffective owing to undocumented implementation issues. In many cases, the degree to which adaptation is feasible without losing effectiveness of the intervention is initially unknown. Rapid iteration and programming adjustments (e.g., via quality improvement research) may contribute to faster generation of knowledge regarding precisely which intervention components should always be maintained.

Translational research studies should consider the relationship between fidelity to “core components” and intervention outcomes [110,143]. In many cases, modification or adaptation of standard approaches (e.g., separation of a single training event into more than one session to accommodate work schedules) aids intervention effectiveness; however, other adaptations (e.g., reduction of training time or content to meet logistical constraints) may hinder effectiveness. While the core components of an intervention are initially unclear, a good practice is to document any adaptations that deviate from the original intervention guidance [144,145]. Such documentation permits thoughtful discussion of whether the adaptation ultimately benefited, altered, or detract from the intervention. Documenting experiences generates knowledge that benefits both local programming and future intervention adaptation for other locations.

#### 3.5.6. Conduct Long-Term Monitoring and Evaluation

Given the often short (less than two-year) measurement of intervention outcomes in many WaSH research projects [146], intervention sustainability is uncertain. The tenets of operational research (Table 2) suggest long-term monitoring can elucidate sustainability factors, disruptions to the system, and potential areas of improvement. Funding allocation may be a strong determinant of the length of monitoring efforts with an eye toward short-term results; however, conserving financial resources means leveraging available data to generate as much value as possible. An intervention could become harmful without investment in proper follow-up evaluation and ethical oversight. Lacking such knowledge and given an assumption of intervention transferability, actors may repeatedly invest in ineffective approaches [147].

Encouraging scientific integrity further recommends replication and peer validation of evaluation findings. Evidence suggests primary and secondary monitoring data may be underused for operational research [148]. Use of open-source data repositories represents good professional practice but is not yet considered or required in many cases. In some instances, privacy, confidentiality, or commercial competition limit data sharing. Compiling and sharing large datasets (if available) and choosing appropriate evaluation methods can enhance statistical certainty, allowing decision makers to consider intervention performance consistency over time and across locations. This may allow analysis of factors of concern, such as novel contaminant classes or social disparities important to sustainable development.

#### 3.5.7. Revisit Results

Replication of effectiveness research can help understand how scale-up alters the initially demonstrated or conceptual efficacy of an intervention. Although not historically as “sexy” or publishable as novel research, replication is a key part of the scientific method used for building theory, illustrating the range of applicability, introducing underrepresented voices, and supporting meta-analysis and systematic review to help synthesize and translate findings for policy and practice. One reason for the gap between research and practice is that limited guidance exists to support structured analyses of gaps in the literature (e.g., evidence maps) prior to project initiation [134]. Actors may assume effectiveness and efficacy research are synonymous, whereas their meanings differ and they require different research methods (Table 2 and Table 3). While efficacy research examines how interventions perform in controlled (including pilot) settings to initially establish face value, effectiveness research seeks to understand how well interventions function when transferred to other real-world applications with altered context.

Few controlled research studies are replicated in more than one setting or context, which reduces confidence in causal inference, the likelihood that findings represent stable effects, the generalizability of results, and the potential for systematic reviews and meta-analyses [46]. Widely differing impacts of the same or comparable interventions for drinking water quality, hand washing, and sanitation have been demonstrated in systematic reviews [149,150,151]. This may indicate a need for more systematically replicating studies across different contexts and settings. Glasgow et al. (2003) recommended placing more research emphasis on how scaling impacts efficacy [152]. One caution for interpreting results is that replication studies may substantially modify a branded intervention (see earlier section “document fidelity and adaptations”) or inadequately address the role of context (see earlier section “consider context”).

#### 3.5.8. Seek Continuing Education in Research Rigor and Reliability

Rigor, reliability, and reproducibility are core considerations for achieving validity in any type of translational research. Rigor refers to thoroughness and accuracy, while reliability and reproducibility refer to stability and consistency. Glasgow and Chambers (2012) recommend translational research methods address rapidity, rigor, transparency, and contextual relevance [153]. Research, practice, and community partnerships may need to explicitly consider and address these different goals and motivations when making decisions, since differences in stakeholder needs are likely to produce tradeoffs for any given methodological option. Theobald et al. (2018) caution that rigor should not be pursued at all costs but must be balanced with practical needs including time frames, budgets, and the probable impacts of the research on decision-making [75].

Journals and other publishers that report scientific studies increasingly develop and adhere to author guidelines, an independent standard, or collectively agreed upon guidance for publications in their field, wherein reproducible, robust, and transparent reporting practices must be met (e.g., International Committee of Medical Journal Editors, 2018 [154]). Still, publication in journals typically occurs once a project is completed, and sponsors and employers may not financially support or require publication in all projects. Sharing methods publicly (e.g., via journals or databases that register study protocols) in advance of a full research report has become more common. If methods are peer-reviewed, this approach contributes to wider relevance and acceptance. Internally documenting an approach and methods likewise provides a foundation to reduce the likelihood of introducing bias in later steps of the research process (e.g., by shifting research questions or selecting data to avoid a null result). Open-access repositories such as the Open Science Framework support sharing as well as transparency and memory throughout all stages of research.

As shown in the comparison of research types (Table 2), traditions and practices may shift over time, necessitating attention to ongoing professional training. Ongoing learning and training resources are relatively prevalent in academic organizations, but are less accessible to those outside academia, such as implementing governments, businesses, or civil society groups [10]. Thus, professionals in these sectors should seek training opportunities in research rigor, and those organizing training opportunities should consider broadening their audience (e.g., via marketing to different actor groups or sharing open online content).

### 3.6. Carrying Out Recommendations in Action: Example of Community-Led Total Sanitation

Community-led total sanitation (CLTS) is a demand-driven, community mobilization intervention originally developed to meet sanitation needs in Bangladesh. The approach has since been widely applied worldwide. This box outlines examples of how best practices in translational research have been used to improve CLTS delivery.

Recognize challenges and potential pitfalls

Despite its popularity, CLTS’ use of shame as a mechanism to mobilize social change has been criticized as unethical, overly coercive, and at times infringing on individual human rights to meet community-wide goals [155,156]. Where communities and implementers are sensitive to evidence of these challenges, CLTS can emphasize positive motivators such as pride and self-determination (Harvey, 2011).

Select a guiding theory and define terms

The Risks, Attitudes, Norms, Abilities, and Self-Regulation model [157] and Integrated Behavioral Model for Water, Sanitation, and Hygiene [158] were developed to model determinants of uptake and practice of WaSH behaviors. These theories have been used to guide studies exploring CLTS and sanitation uptake across different contexts [159,160,161]. Application of theories with well-defined constructs allows findings to be generalized and compared across settings, facilitating future studies of sanitation uptake.

Consider context

Since its development in Bangladesh, CLTS has been widely applied elsewhere. However, these applications have not consistently reproduced similar improvements in sanitation coverage. Contextual factors can explain some of these differences. Harter et al. (2018) found that strong social cohesion, trust, and inclusiveness facilitated CLTS [159]. Crocker et al. (2017) found that CLTS effects were more likely to be sustained in villages with higher poverty and initial open defecation rates and less exposure to other WaSH projects [162]. These findings align more closely with the conditions for which CLTS was originally designed [163] and suggest that translational research may be applied to understand whether a given intervention is likely to be appropriate for a particular context.

Involve diverse stakeholder groups

CLTS is, at its heart, a community-driven approach. Facilitators adapt activities to the local context and identify “natural leaders” to champion efforts from within the community. When implemented as intended, a skilled CLTS facilitator allows communities to develop an action plan that suits their needs. Co-generation of knowledge with local communities helps promote development of solutions that are better suited to community needs, and participatory workshops in which facilitators share local learnings across organizations have helped promote innovation [164].

Document fidelity and adaptations

In a study of seven countries [147], CLTS triggering techniques had been adapted in all settings. Some adaptations were made to improve implementation or effectiveness outcomes (e.g., omitting a triggering activity that reduced participation rates), while others were made for non-evidence-based reasons (e.g., omitting a triggering activity that made facilitators uncomfortable). Planned, evidence-based adaptations generally improved CLTS quality, while adaptations for convenience or other non-evidence-based reasons often vitiated the CLTS theory of change. Translational research to understand context and the nature and effects of adaptations can identify when and where adaptations are necessary and appropriate. Translational research can also help scale and adapt approaches to new contexts. Studies of sanitation delivery and uptake drivers in urban environments have led to successful adaptation of the CLTS approach, which was originally designed for rural settings, to the urban environment [165,166].

Conduct long-term monitoring and evaluation

The goal of CLTS is to achieve open-defecation free (ODF) communities, and CLTS activities often cease once communities are declared ODF. However, results from long-term monitoring studies suggest that ODF communities often revert to open-defecation in the years following ODF certification. Studies conducting long-term monitoring have attempted to explain the factors involved in sustaining CLTS results [162,167].

Revisit your results

Cameron et al. (2019) conducted a randomized trial of CLTS delivered at scale in rural East Java, Indonesia. In the scaling process, local government took on the role of implementation, previously headed by non-governmental organizations [168]. While small-scale pilot tests of NGO-implemented programs significantly improved sanitation coverage and infectious disease and anthropometric outcomes in children, government-implemented CLTS had no effect at scale.

### 3.7. Examples and Resources for Carrying Out Translational Research Recommendations

Example studies that define guiding theory and terminology, as follows:

- Koehler et al. (2018) carefully articulated how cultural theory, in the context of rural water management, provided a basis for understanding the cooperation and conflict between management cultures to handle operational, financial, institutional and environmental risk. The authors thoroughly defined terminology relevant to cultural theory and a pluralist framework at the outset of the study [169].
- Bresee et al. (2016) explained how theory informed their study design in multiple ways. Researchers aligned focus group data collection methods with diffusion of innovation theory, and based data analysis methods on grounded theory. They cited the original works that provided a basis for these approaches when reporting [107].

Guidance for considering context, as follows:

- Pfadenhauer et al. (2017) developed the Context and Implementation of Complex Interventions (CICI) framework for use as a determinant or evaluation framework broadly applicable to public health interventions. They describe contextual domains but do not define constructs within domains [87].
- The Consolidated Framework for Implementation Research (CFIR) outlines contextual determinants likely to influence implementation [70] and provides qualitative and quantitative tools for researchers to assess context (https://cfirguide.org/).
- Craig et al. (2018) proposed guidelines for addressing the influence of context when adapting and scaling population health interventions [132].

Example studies that consider context, as follows:

- West et al. (2016) investigated the effects of context on residential recycled water schemes in Australia to assess common reasons for failure. They found contextual factors such as regulatory environment, operational costs, and consumer preferences to influence success [170].
- Novotný et al. (2018) conducted a systematic review to identify which studies reported the contextual factors associated with sanitation adoption and implementation. They proposed a typology that categorizes contextual factors into societal, community, interpersonal, and individual levels [171].
- Amjad et al. (2016) used service provider interviews to assess perceived willingness and ability as measures of actor readiness for implementing the WHO’s Water Safety Plan intervention [172]. Setty et al. (2019a) used this preliminary research on context and the CFIR to inform a multi-criteria decision process for selecting appropriate intervention guidance for US source water risk management [173]. The authors recommended adapting the intervention guidance, e.g., by hybridizing with existing programs, to fit the context.

Example studies that attend to stakeholder inclusion, as follows:

- White et al. (2008) qualitatively analyzed data from in-depth interviews with water managers to assess perceptions. They published a model for boundary organizations to mediate negotiations between scientific and political actors to inform decision making for water resources [16].
- Liu et al. (2008) applied an integrated modeling framework that engages stakeholders in every step from problem formation to monitoring and evaluation. They suggest adoption of research results and informed decision making requires open lines of communication among all parties associated with the project [174].
- Nelson et al. (2014) suggest the perspectives and values of stakeholders must be considered in the process of policy formation and decision-making. They examine the strategy of assigning roles and responsibilities to different stakeholders, concluding that engagement between actors and ‘clear messages’ from top level political entities are important factors in the provision of sanitation facilities [175].

Example studies that measure fidelity and document adaptations, as follows:

- Sigler et al. (2014) helped to identify core components of community-led total sanitation by documenting (a) which behavior change frameworks and techniques were commonly used, (b) how implementation differs by region and context, and (c) which implementation activities were considered most valuable in achieving and sustaining desired intervention outcomes [176].
- Crocker et al. (2016) evaluated how an implementation variation to train natural leaders (motivated community members) to influence peer behaviors affected implementation and intervention outcomes for community-led total sanitation [177].
- Benjamin-Chung et al. (2017) assessed program delivery of a UNICEF intervention called Sanitation Hygiene Education and Water Supply in Bangladesh (SHEWA-B) to 20 million rural people to assess whether implementation quality affected program success, as measured by a survey of household recall. They developed an implementation quality index and compared fidelity measures, such as hygiene promotor visitation and knowledge of key messages, to outcome measures, such as health behaviors and sanitation infrastructure access [109].

Example studies that examine long-term trends, as follows:

- Using annual school-level data approximately 14 years after implementation of a sanitation intervention in India, Adukia (2017) demonstrated health benefits associated with school-latrine construction (especially for younger children) but not educational benefits [178].
- Cronk and Bartram (2017) aggregated existing country-level monitoring program data across Nigeria and Tanzania to develop Bayesian network models comparing diverse water system and management types. By following systems ranging from zero to more than 30 years of use, they garnered insights on water system functionality and service availability [113].
- Following a national program adopted in 2011, Senbeta and Shu (2019) studied three different management modalities (community, local government, and other external sources including non-governmental organizations) used for rural water supply sustainability in Ethiopia. Data were collected via household surveys, interviews with committee members, direct observation, and records review. They found community-management modalities performed better on most sustainability indicators [179].

Examples of replication studies, as follows:

- The WaSH Benefits randomized controlled trials in Kenya and Bangladesh assessed the effects on stunting and diarrhea of WaSH interventions separately and in combination with nutrition interventions. Interventions were adapted to fit the local cultural context but adhere to the same theory of change [103,180].
- Setty et al. (2017) replicated an earlier study on risk management of non-chlorinated groundwater supplies in Iceland [181,182]. It assessed water quality and public health outcomes of the WHO Water Safety Plan intervention as applied to chlorinated surface water supplies in France and Spain.

Resources for maintaining and improving research rigor and reliability, as follows:

- The Open Science Framework (https://osf.io/) is a free scholarly commons that seeks to align practice with scientific values by improving research openness, integrity and reproducibility. They also offer a training curriculum (https://osf.io/48up3/).
- Dataverse (https://dataverse.org/) offers an open-source research data repository.
- The US National Institutes of Health provide training modules on rigor (https://www.nih.gov/research-training/rigor-reproducibility/training) as well as periodic email updates on rigor and reproducibility topics (https://www.nih.gov/research-training/rigor-reproducibility).
- The Center for Effective Global Action at the University of California, Berkeley offers in-person fee-based Research Transparency and Reproducibility Training (RT2) events in Los Angeles and London, and openly shares the training materials.

Chambers’ 2017 text “Can we know better? Reflections for development” discusses achievement of rigor within complex interventions for those working toward the SDGs.

### 3.8. Limitations and Remaining Research Needs

This paper offers a starting point for adapting translational research methods to address WaSH needs. It was not intended to provide absolute or exhaustive classification but rather a springboard for debate and concept development. It recommends increased attention to cooperation, communication, and coordination among WaSH actors to facilitate efficient translation between research and practice. The recommendations address improved study design and reporting to clarify understanding and lessons learned about the complexity of WaSH interventions and scale-up to meet public needs. As the number of people using comparable methods for translational research on WaSH grows in the future, it may be increasingly possible to use broad, consensus-based exercises, meta-analyses, and scoping or systematic reviews to compare practices at different scales. Translational research methods will likely require further adaptation to enable application to diverse sub-fields of public health, environmental science, and WaSH. Future studies could use demand-driven, stakeholder-inclusive approaches to develop guidance on common translational research methods, models, or frameworks for WaSH professionals.

## 4. Conclusions

With increasing pressure to incorporate evidence into decision-making and the SDG deadline of 2030 fast approaching, research plays a valuable role in informing WaSH policy and practice [12]. WaSH professionals are largely working toward similar goals, led by overarching global development guidance [183]. Improved understanding of the terminology, classification, and guidance available for translational research types, such as quality improvement, could promote quicker translation of evidence into action and help to optimize services provided by professional actors.

Based on this study, recommendations for enabling translational research to bridge research and practice include:

- Recognizing challenges and potential pitfalls,
- Selecting an appropriate theoretical basis for study design and defining terms,
- Considering the role of context,
- Involving a diverse set of stakeholders throughout research phases,
- Documenting intervention adaptations,
- Supporting follow-up monitoring and evaluation,
- Understanding the difference between efficacy and effectiveness research and actively replicating studies in more than one setting or context, and
- Seeking ongoing training in research methods and rigor.

Translational research should consider the needs of researchers and other actors [10,184]. To effectively increase engagement in translational research processes, actors must communicate and respect each other’s contributions (e.g., service providers’ and receivers’ intimate knowledge of field conditions and complexities; researchers’ needs for ethical oversight and high-quality methods, data, and analysis). Fostering effective knowledge translation systems requires active “boundary management” between and among affected groups, including communication, translation, and mediation [12,15]. This can be accomplished within the framing of translational research processes. Setting expectations for an individual, group, or institution to serve as facilitators may help bridge the gap between scientific evidence and WaSH services.

## Figures and Tables

**Table 1:** Principal roles of water, sanitation, and hygiene actors in translational research ^1^.

Roles in Translational Research						
Individual or Collective Actors ^2^	Fund Research	Lead Research Investiga-tions	Interpret and/or Communicate Research	Provide Policies, Guidance, Technical Assistance, and/or Facilitation (“Decision Makers”)	Implement Practices (“Service Providers”)	Receive Services/Engage in Behaviors
Professional Actors						
Researchers		X	X			
Educators			X			
Governments	X	X	X	X	X	
Multilateral organizations	X	X	X	X	X	
Service providers		X	X		X	
Civil society organizations		X	X		X	
Policymakers	X		X	X		
Businesses	X	X	X		X	
Foundations	X		X			
Networks	X		X			
Media			X			
Non-Professional Actors						
Volunteers		X	X			X
General Public			X			X

^1^ Examples are representative but not exhaustive; other outcomes are possible. ^2^ Some individuals or groups may fit in more than one actor category.

**Table 2:** Principal translational research types relevant to water, sanitation, and hygiene and their major characteristics.

Research Type (Related or Commonly Used Associated Terms)	Purpose	History/ Origins	Primary Actors	Good Practices	Resources for More Information
Pure (fundamental, basic)	Observe, describe, question, hypothesize, experiment, and develop theory	Scientific method (17th century)	Researchers	Expand knowledge; avoid bias; share findings among scientific community to develop evidence base	Scientific Method in Brief [40]; Qualitative Research Methods: Collecting Evidence, Crafting Analysis, Communicating Impact [41]
Efficacy (comparative efficacy)	Determine if an intervention produces desired results in controlled or limited settings	18th–20th centuries; randomized design (e.g., Peirce, Fisher) [42,43,44]	Researchers	Systematic experimentation or observation; avoid confounding (e.g., via blinding, matched controls, randomization); offer measure of statistical certainty	Improving the reporting of pragmatic trials: An extension of the CONSORT statement [45];
Effectiveness (comparative effectiveness)	Determine if an intervention produces desired results in diverse real-world settings	18th–20th centuries; randomized design (e.g., Peirce, Fisher) [42,43,44]	Researchers	Systematic experimentation or observation; understand confounding; offer measure of statistical certainty	Study designs for effectiveness and translation research: identifying trade-offs [46]; The road to results: designing and conducting effective development evaluations [47]
Applied ^1^	Apply science with intention to address a practical problem	18th–20th centuries; especially post-WWII	Researchers; service providers; decision-makers	Problem- or client-driven; may or may not lead to invention	Cycles of invention and discovery: Rethinking the endless frontier [48]; Routledge Handbook of Water and Health [49]
Diffusion	Examine how novel ideas and practices (often passively) spread among groups of people	Rogers (1962) Theory of Diffusion of Innovation	Researchers; service providers; decision-makers	Classify adopters by stages; study channels, rates, variables (e.g., member characteristics), and consequences	Diffusion of innovations, 5th edition [50]; Social networks and health: A systematic review of sociocentric network studies in low- and middle-income countries [51]
Dissemination	Target (often active) distribution of information or an intervention to (a) specific audience(s)	Rogers (1962) Theory of Diffusion of Innovation; government initiatives beginning in late-1990s and early-2000s (e.g., US National Institutes of Health)	Researchers; service providers; decision-makers	Identify potential adopters; facilitate uptake and effective use of specific (implicitly evidence-based) interventions	An overview of research and evaluation designs for dissemination and implementation [32]
Implementation (knowledge translation)	Develop methods to promote uptake of research and evidence-based practices into routine practice	Dedicated *Implementation Science* journal begun in 2006, following evidence-based movement of 1990s and recognition of science-to-service gap	Service providers; researchers (as external facilitators)	Grounded in theory; trans-disciplinary research teams; evidence-based practice, barrier and strategy selection; documenting context and adaptations	An introduction to implementation science for the non-specialist [24]
Operations/ operational (analytics, systems analysis, management science)	Apply scientific principles to business management, providing quantitative basis for complex decisions	Babbage (1840s); military planning during World Wars I and II (early-mid 1900s) [52]	Service providers; researchers	Often uses simulation, modeling, mathematical, or statistical techniques; practical objective to optimize complex systems	Encyclopedia of operations research and management science [53]; Structured Operational Research and Training IniTiative (SORT IT) from Médecins Sans Frontières (https://msf.lu/de/node/283)
Participatory action (action, community-based participatory, transdisciplinary)	Undertake self-reflective enquiry to solve social problems, create change to improve quality of life	Popular education (1930s) [54]; Lewin’s (1946) work on action research and minority problems [55]; theories of transdisciplinary knowledge co-production [36,56]	Civil society, service providers, community organizations, and/or public (acting as researchers); researchers	Equitable involvement; shared decision-making and ownership; critical self-examination; empower participants to develop and implement improvements	Action research, 4th ed. [57]; Methods in community-based participatory research for health [58]; Community-based participatory research as a tool to advance environmental health sciences [59]; Michigan Public Health Training Center online course “CBPR: A Partnership Approach for Public Health” (https://www.mitrainingcenter.org/courses/cbprs0218)
Quality improvement (improvement science)	Design and trial strategies to improve specific problem within specific system	Deming (1950s) [60]; Institute for Healthcare Improvement (IHI) Model for Improvement (2003)	Service providers; researchers (as external facilitators)	Create effective change by repeating plan-do-check-act (PDCA) or plan-do-study-act (PDSA) cycles	The improvement guide [61]; Public Health Foundation (phf.org)

^1^ Applied” may serve as a distinct research type (in contrast with pure research) or an umbrella descriptor inclusive of multiple translational research types.

**Table 3:** Examples of water, sanitation, and hygiene studies addressing various translational research types and questions ^1^.

Research Type	Model Research Question (Linked to End Goal/Outcome)	Example Studies	Specific Research Questions
Pure	How can we understand a phenomenon? Can we measure ___? Does evidence support theory? How are phenomena related?	Virus inactivation mechanisms: Impact of disinfectants on virus function and structural integrity [99] Defining endemic cholera at three levels of spatiotemporal resolution within Bangladesh [100]	How are viral functions affected by exposure to inactivating treatments? What does whole-genome sequencing tell us about cholera diversity and transmission dynamics at individual, household, regional and intercontinental scales?
Efficacy	Does intervention work in a controlled setting or specific population?	The effects of input materials on ceramic water filter efficacy for household drinking water treatment [101] A community-designed play-yard intervention to prevent microbial ingestion: A baby water, sanitation, and hygiene pilot study in rural Zambia [102]	How do production methods and quality control protocols alter efficacy of ceramic drinking water filters? Did a novel play-yard intervention help rural farming families reduce infant and young children’s exposure to human and free-range livestock feces?
Effectiveness	Does intervention work in real-world settings?	Effects of water quality, sanitation, handwashing, and nutritional interventions on diarrhea and child growth in rural Bangladesh: a cluster randomized controlled trial [103]	Do individual water, sanitation, handwashing, or nutrition interventions reduce linear growth faltering? Are combined water, sanitation, and handwashing interventions more effective at reducing diarrhea than individual interventions? Does the combination of water, sanitation, handwashing, and nutrition interventions reduce growth faltering more than each individual intervention?
Applied	What type of intervention might solve a problem? Does intervention fulfill an identified need? Should intervention be modified?	Characterization of pit latrines to support the design and selection of emptying tools in peri-urban Mzuzu, Malawi [104]	What characteristics of household pit latrines are important when designing and selecting pit latrine-emptying tools?
Diffusion	How or why does intervention spread (often passively)? Who carries information?	Exploring the utility of diffusion theory to evaluate social marketing approaches to improve urban sanitation in Malawi [97] Risk management for drinking water safety in low and middle income countries–cultural influences on water safety plan (WSP) implementation in urban water utilities [105]	Do ‘first movers’ display characteristics of innovators including relatively high incomes and risk-taking behaviors? What is the role of interpersonal information sources (opinion leaders and change agents) on the decision-making process of ‘first movers’ of the ecological toilet? Do ‘first movers’ report all five attributes described by Rogers (2003) [50] (relative advantage, compatibility, simplicity, observability and trialability) as positive reasons for purchasing the ecological toilet? How do cultural constructs in different countries influence implementation of the water safety plan intervention?
Dissemination	How can we (often actively) facilitate spread of an intervention? Who or what influences spread of the intervention?	Factors supporting the sustained use of solar water disinfection—experiences from a global promotion and dissemination program [106] Social network targeting to maximize population behaviour change: A cluster randomized controlled trial [98] “A child is also a teacher”: exploring the potential for children as change agents in the context of a school-based WASH intervention in rural Eastern Zambia [107]	Which factors influenced acceptance and sustained use of a solar water disinfection intervention? Which methods of targeting influential individuals produce the greatest cascades or spillover effects? Can children can influence their families to adopt healthy WASH behaviors in Eastern Zambia? If so, how?
Implementation	How can we scale an evidence-based intervention effectively?	Teachers and sanitation promotion: an assessment of community-led total sanitation in Ethiopia [108] Scaling up a water, sanitation, and hygiene program in rural Bangladesh: the role of program implementation [109] Sustainability and scale-up of household water treatment and safe storage practices: enablers and barriers to effective implementation [110]	How did context and process (implementation arrangements) influence effectiveness of the community-led total sanitation intervention? How did the implementation process influence achievement of desired intervention outcomes? What are the barriers and enablers for sustaining and scaling up household water treatment and safe storage practices such as boiling, chlorination, and filtration?
Operations/ Operational	How can we better understand and intervene on the factors affecting ongoing operational or systems processes?	Bisphenol-A removal in various wastewater treatment processes: Operational conditions, mass balance, and optimization [111] Determinants of stunting in Indonesian children: evidence from a cross-sectional survey indicate a prominent role for the water, sanitation and hygiene sector in stunting reduction [112] Factors influencing water system functionality in Nigeria and Tanzania: a regression and Bayesian network analysis [113]	Which operational conditions promoted BPA removal during wastewater treatment? What variables were statistically associated with stunting in Indonesian children? What were the data relationships between water system functionality and poverty, population density, groundwater availability, and distance to urban centers?
Participatory action	How can we work collectively to create knowledge to address a problem?	Improving community health through marketing exchanges: A participatory action research study on water, sanitation, and hygiene in three Melanesian countries [114] Action research for sustainable water futures in western Sydney [115] Using photovoice as a community based participatory research tool for changing water, sanitation, and hygiene behaviours in Usoma, Kenya [116]	How did impoverished communities in urban and peri-urban areas attempt to meet their water, sanitation, and hygiene needs through marketing exchange? How do people who are ‘hard to reach’ or seen as ‘apathetic’ engage using an ‘action-conversations’ tool that explores the social climate for action? How can scientific or technical messages be framed in the language of the community? What are local perceptions and practices around water-health linkages? Did photovoice tool help participants actively engage and/or change their behaviors?
Quality improvement	How can we adjust systems to achieve a desired improvement?	Strengthening healthcare facilities through water, sanitation, and hygiene improvements: A pilot evaluation of ‘‘WASH FIT’’ in Togo [117] Implementation science in low-resource settings: Using the interactive systems framework to improve hand hygiene in a tertiary hospital in Ghana [118]	How does the continuous improvement tool work? What are the implementation outcomes? Is the tool acceptable and feasible? Can a quality improvement intervention improve hand hygiene compliance in low-resource settings?

^1^ Few, diverse examples are provided here. Other appropriate examples may be available for a given research type or water, sanitation, and hygiene topic.
